# Reported Theory Use by Digital Interventions for Hazardous and Harmful Alcohol Consumption, and Association With Effectiveness: Meta-Regression

**DOI:** 10.2196/jmir.8807

**Published:** 2018-02-28

**Authors:** Claire Garnett, David Crane, Jamie Brown, Eileen Kaner, Fiona Beyer, Colin Muirhead, Matthew Hickman, James Redmore, Frank de Vocht, Emma Beard, Susan Michie

**Affiliations:** ^1^ Department of Behavioural Science and Health University College London London United Kingdom; ^2^ Department of Clinical, Educational and Health Psychology University College London London United Kingdom; ^3^ Institute of Health and Society Newcastle University Newcastle upon Tyne United Kingdom; ^4^ School of Social and Community Medicine University of Bristol Bristol United Kingdom

**Keywords:** alcohol drinking, behavior, addictive, regression analysis, meta-analysis, randomized controlled trial, Internet

## Abstract

**Background:**

Applying theory to the design and evaluation of interventions is likely to increase effectiveness and improve the evidence base from which future interventions are developed, though few interventions report this.

**Objective:**

The aim of this paper was to assess how digital interventions to reduce hazardous and harmful alcohol consumption report the use of theory in their development and evaluation, and whether reporting of theory use is associated with intervention effectiveness.

**Methods:**

Randomized controlled trials were extracted from a Cochrane review on digital interventions for reducing hazardous and harmful alcohol consumption. Reporting of theory use within these digital interventions was investigated using the theory coding scheme (TCS). Reported theory use was analyzed by frequency counts and descriptive statistics. Associations were analyzed with meta-regression models.

**Results:**

Of 41 trials involving 42 comparisons, half did not mention theory (50% [21/42]), and only 38% (16/42) used theory to select or develop the intervention techniques. Significant heterogeneity existed between studies in the effect of interventions on alcohol reduction (I^2^=77.6%, *P*<.001). No significant associations were detected between reporting of theory use and intervention effectiveness in unadjusted models, though the meta-regression was underpowered to detect modest associations.

**Conclusions:**

Digital interventions offer a unique opportunity to refine and develop new dynamic, temporally sensitive theories, yet none of the studies reported refining or developing theory. Clearer selection, application, and reporting of theory use is needed to accurately assess how useful theory is in this field and to advance the field of behavior change theories.

## Introduction

Excessive alcohol consumption is a major avoidable risk factor for the global burden of disease and injury [1]. An estimated 4.9% of the world’s adult population suffers from alcohol use disorders [2], with alcohol causing an estimated 3.8% of all deaths and 4.6% of disability-adjusted life years [1]. Alcohol-related costs amount to more than 1% of the gross national product in high- and middle-income countries [1]. Digital behavior change interventions are products or services to promote behavior change delivered via computer technology, which includes smartphone apps, websites, computer programs, wearable devices, and telecommunications [3]. Digital behavior change interventions have a potentially broader reach than face-to-face brief interventions and have been identified as cost-effective [4]. Reviews of digital interventions to reduce hazardous and harmful alcohol consumption have found that they can be more effective than controls [4-14]. However, there is substantial heterogeneity between the effectiveness of different interventions that is currently unexplained [5,8,9,11,12].

Behavior change theories “explain why, when and how a behaviour does or does not occur, and the important sources of influence to be targeted in order to alter the behaviour” [15]. A good theory should have a “parsimonious, coherent explanation of phenomena” [15] that is comprehensible, internally consistent, generates testable predictions, and is not contradicted by observations [16]. Theories often lack specificity and so fail to generate hypotheses that can be tested in the real world [17]. These testable predictions can and should be used as a basis to refine and improve theories [18] or to retire theories that do not explain or predict intervention outcomes [16]. Using a good behavior change theory in intervention development and evaluation has many potential benefits [19]. Theories can inform researchers about which variables might be most influential in mediating the target behavior, thereby providing a systematic method for selecting [20] and refining appropriate intervention techniques [21]. Using a theoretical framework for data collection means that evidence of effectiveness can be accumulated across different contexts, populations, and behaviors [19], and the process of adapting and refining interventions is more efficient [20]. Theory-based interventions can reveal what makes an intervention effective by enabling empirical tests of theoretical propositions. These can, in turn, provide a basis for refining theory [18], and future theory-based interventions are likely to be improved [22]. This illustrates the concept of a virtuous spiral between theory and intervention development whereby theory can inform intervention development, and interventions can test and refine the underlying theory [3]. These benefits suggest that systematic use of a high quality behavior change theory in intervention development may result in a more effective intervention [19,23-25] and be able to inform future interventions [22].

The extent to which theory is used may explain some of the substantial heterogeneity found between the effectiveness of different digital alcohol reduction interventions. Mixed evidence exists with both positive [21,26-30] and negative associations [31,32] being found between theory use and the effectiveness of behavior change interventions. There are a number of factors that may contribute to this pattern of results. The value of theory is dependent on using a high quality and appropriate theory that is relevant to the behavior [15]. Furthermore, the way theory has been used and reported is generally inadequate; many studies do not report theory use in intervention development or evaluation [23,30,33-36]. If interventions are described as having a theoretical basis, this description is often unclear or not extensive [24,29]. For example, a review of physical activity and dietary interventions found that only half reported using theory and, of those, only a small proportion systematically applied theory [29].

The theory coding scheme (TCS) is a tool used to describe the theoretical basis of interventions [19]. The tool was specifically developed to inform evidence syntheses and has been widely used in meta-analyses to assess the potential association between theory and intervention effectiveness [14,25,29]. The TCS has 19 items—each with satisfactory interrater reliabilities—that can be grouped into six categories of theory use: reference to underpinning theory, targeting of relevant theoretical constructs, using theory to select recipients or tailor interventions, measurement of constructs, testing of theory, mediation effects, and refining theory [19]. Composite scores can provide an estimate of the extent of reported theory use, which also facilitates the assessment of whether an association exists between the extent of theory use and intervention effectiveness [29].

The association between theory use in computer-delivered interventions and alcohol-related outcomes in the general population has been assessed using meta-regression [14]. This review found no association between the extent of theory use in intervention development and effectiveness but did find that the use of a particular theory—the social norms approach [37]—was associated with improved outcomes [14]. This paper will investigate whether these findings generalize to populations of hazardous and harmful drinkers. This population is of particular interest because they experience more economic, health, and social costs compared with low-risk drinkers [38]. There is also a need for replication of studies, including meta-analyses, to confirm initial findings and build a strong evidence base [39,40].

This paper reports a theoretical analysis of interventions that we planned as part of a Cochrane review of the effectiveness of digital interventions for reducing hazardous and harmful alcohol consumption in community-dwelling populations (systematic review registration number: CRD42015022135) [41,42].

This paper will address the following research questions:

1. How is theory use reported in the development and evaluation of digital interventions for reducing hazardous and harmful alcohol consumption?
  - Which items and categories of theory use are used most frequently?
  - What is the extent of reported theory use (mean total theory use score)?
2. Is there an association between intervention effectiveness and items, categories, and extent of theory use?

## Methods

### Search Strategy and Study Selection

Studies for inclusion in the systematic review were identified through a broad search of databases (eg, MEDLINE, Cochrane Library, CINAHL, PsycINFO, and Clinicaltrials.gov) and relevant websites (eg, International Alcohol Information Database, Beacon 2.0, and Drug and Alcohol Findings). The reference lists of all included studies and relevant reviews were checked. The search combined terms for hazardous or harmful alcohol consumption (eg, alcohol, drinking, alcohol use, and risks) with terms for computer-assisted therapy or digital interventions (eg, Internet, computers, and smartphone). Full details of the search strategy are reported in the protocol in the Cochrane Library [41].

### Inclusion Criteria

Studies were randomized controlled trials with the outcome measure of quantity of alcohol consumed (in grams per week), which could be reported in standard drinks, alcohol units, or similar. Participants were community-dwelling individuals who could have been recruited in a range of settings (eg, primary health care, social care, educational, and workplace) and were under no obligation to complete the intervention (eg, mandated college students). Participants were screened and identified as hazardous or harmful drinkers typically via completing short online questionnaires such as the Alcohol Use Disorders Identification Test (AUDIT) or quantity-frequency measures. The intervention had to target alcohol consumption or alcohol-related problems in the drinker and be delivered primarily through a digital device. A comparator condition must have been included (eg, no intervention, usual care, feedback or general health advice, or health information via printed leaflets or booklets). Full details of the inclusion criteria are in the protocol [41].

### Review Procedure

The review procedure consisted of two phases to identify relevant studies using the inclusion criteria detailed above. Initially, studies were reviewed independently by two researchers based on their title and abstract, using Endnote to ensure consistency. A conservative approach was taken so that studies were included if their relevance to the review was uncertain. In the second phase, the full research paper of any studies identified as potentially eligible were reviewed independently by two researchers. Any discrepancies were resolved by discussion and by consulting a third researcher if necessary. The inclusion criteria were amended to reflect any clarifications that occurred during the discussion of discrepancies.

### Data Extraction

A standardized data extraction form was developed and piloted, which two researchers used to independently carry out data extraction of all included studies. Data were extracted about the following: details of the intervention (eg, setting, duration, size, and characteristics of sample) and baseline and follow-up data for the primary outcome measure of the main Cochrane review (grams of alcohol consumed per week).

A theoretical analysis of the studies was conducted using the TCS [19]. Coding extended to any development, feasibility, or protocol papers that were referenced in the included studies and may have reported supplementary information about the intervention. Two researchers independently coded a sample of 5 studies using the TCS. Differences were resolved through discussion, and a third researcher was consulted if agreement was not reached; notes on the coding guidelines were made accordingly. Four further rounds of testing were performed until the interrater reliability (IRR) reached a substantial level of agreement (prevalence-adjusted bias-adjusted kappa, PABAK statistic greater or equal to .70 [43]). The PABAK statistic was .84 across the five rounds of IRR checking, which reflects a substantial level of agreement. After this level of agreement was achieved, the remaining studies were coded by one researcher.

The TCS was amended for use in this systematic review; two items (“quality of measures” and “randomization of participants to condition”) were excluded because they related to methodological issues rather than informing whether or how theory was used in an intervention. The amended TCS had 17 items (three of which had subitems); see Table 1 for a list of these items and their descriptions. Each study was dummy coded for the TCS items as present (1) or absent (0). If any theory was mentioned (item 1), then the relevant name was documented, regardless of whether empirical support for the theory existed. If a protocol or other paper was referenced as describing the intervention, then that paper was also coded for those items relating to intervention development (items 1-11). Composite scores were calculated for the six categories of theory use (see Table 2 for a description of these categories) and a total theory use score [19]. The total theory use score was a sum of all 17 items, three of which had subitems, which resulted in a maximum possible score of 22. For the composite scores, any item detailing “all” (items 7 and 8) that was coded as present was also coded as present for the equivalent item detailing “at least one” (items 10 and 11) so that the composite scores were representative (as in [25]).

### Analysis

Frequency counts and descriptive statistics were used to describe the theoretical basis to digital interventions for alcohol reduction. The range and frequency of theories used were tabulated.

The meta-regressions were conducted in Stata (StataCorp; version 14). Effect sizes were based on a random effects model, as the intervention effects were likely to have residual heterogeneity not modeled by the covariates. The effectiveness of the intervention was measured using the primary outcome measure of difference in grams of alcohol consumed per week between the digital intervention and control arms at the longest follow-up time point. The weighted mean difference method was used to estimate pooled effect sizes and 95% CIs. Previous simulation studies have found that for accurate estimates in meta-regression, at least 40 studies are required [44] and that more than 200 studies are required for 80% power to detect modest associations [45].

A series of unadjusted random effects meta-regression analyses were conducted to examine the association between the TCS covariates (individual theory items, included by at least 10% (5/42) of studies; the categories of theory use; and total theory use) with intervention effectiveness and the percentage of the between-study heterogeneity (adjusted R^2^) explained by each predictor. The regression coefficient (B) represented the mean of the unstandardized effects between trials that differentially included each TCS covariate. A negative coefficient for a covariate indicated that the studies reporting that theory item, or with higher composite scores for the categories of theory use and total theory use, were associated with a larger reduction in consumption than studies that did not.

To investigate the independent associations, an adjusted meta-regression analysis was conducted, including all of the variables that had a meaningful association with intervention effectiveness in the unadjusted models. A meaningful association was defined as B>23, based on the lower boundary of a 95% CI for the effect found in a systematic review of brief alcohol interventions [46].

In the event of a nonsignificant result, a Bayes factor was calculated to establish the relative likelihood of the null versus the experimental hypothesis given the data obtained. The experimental hypothesis was that the TCS covariate was associated with intervention effectiveness, and the null hypothesis was that there was no association. The Bayes factors were calculated with the alternative, directional hypotheses conservatively represented in each case by a one-tailed, nonuniform distribution using the online calculator associated with Dienes [47,48]. The standard error was specified as the expected effect size (ie, 23), which means plausible values have been effectively represented between 0 and twice the effect size, with smaller values more likely. Bayes factors allow the distinction between two interpretations of a null result: there is evidence for the null-hypothesis or that the data are insensitive in distinguishing an effect. Bayes factors vary from 0 to ∞: values of 3 to 10 indicate moderate evidence for the experimental hypothesis over the null, whereas values greater than 10 indicate strong evidence; values of 0.10 to 0.33 indicate moderate evidence for the null over the alternative, whereas values less than 0.10 indicate strong evidence; and values between 0.33 and 3 indicate that the data are insensitive in distinguishing an effect [49].

## Results

### Study Selection

**Figure 1:**
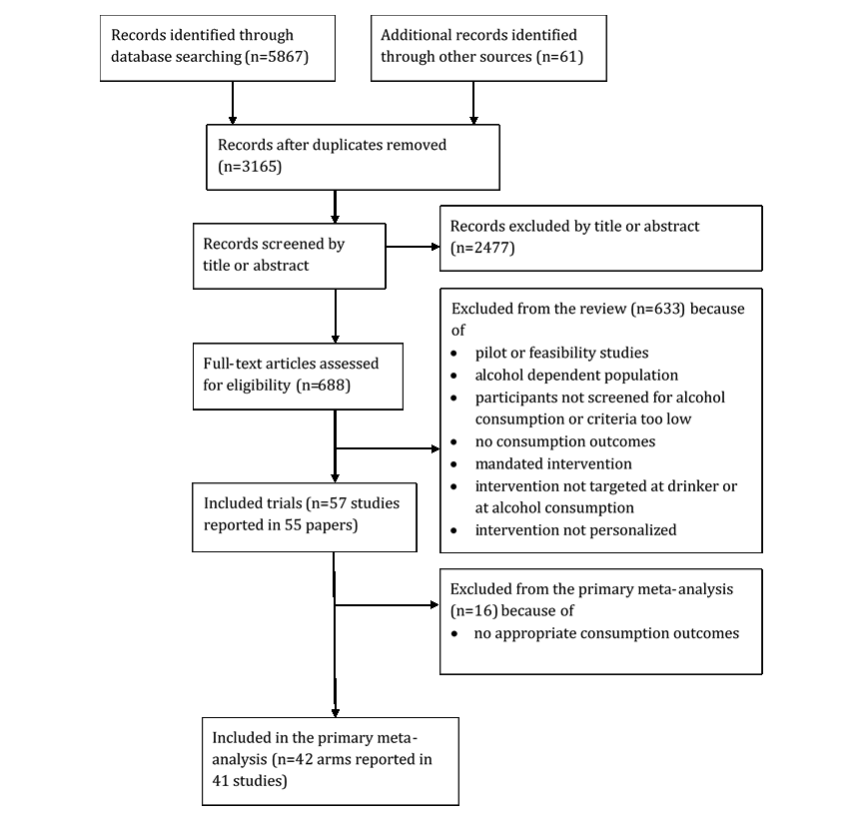
Flowchart showing identification of included trials (reproduced from the main Cochrane review).

Studies were selected for this meta-regression if they were included in the primary meta-analysis of the Cochrane review [42]. A total of 5928 records were identified through database searching and through other sources, with 3165 records remaining after duplicates were removed. Records were then screened by their title and abstract (with 2477 excluded) before the full text was screened (633 excluded; see Figure 1 for reasons for exclusion). Forty-one trials compared a digital intervention (one contained two digital arms) with a control (these included assessment only, waiting list control groups, and provision of standard health-related information) and reported appropriate information for inclusion in the primary meta-analysis. This resulted in 42 digital intervention arms. Multimedia Appendix 1 reports the references to studies included in this meta-regression.

### Study Characteristics

The 42 digital intervention arms included 19241 participants (9631 randomized to a digital intervention and 9610 randomized to a control condition). The longest period of follow-up ranged from 1 month (n=8) to 12 months (n=7). Interventions were Web-based in 34 studies, comprised a stand-alone computer program in 6 studies, and a smartphone app in 1 study. A total of 24 studies focused on students or younger adults (<25 years), whereas the others recruited adults of any age. Use of the intervention was restricted to a specific location (eg, primary care clinic or psychology lab) in 10 studies, and 30 trials allowed participants to use the intervention at the location of their choice. The majority of the studies (n=23) took place in North America, 9 took place in continental Europe, 4 in Scandinavia, 2 in the United Kingdom, 2 in New Zealand, and 1 in Australia.

### How Is Theory Use Reported in Digital Interventions?

Table 1 reports the frequency of reporting in studies for the TCS items, and Table 2 reports the composite scores for the six categories of theory use and the total use of theory. The most frequently reported theory items were as follows: “theory or model mentioned” (50% [21/42]), “targeted constructs mentioned as a predictor of behavior” (40% [17/42]), and “theory or theoretical predictors used to select or develop intervention techniques” (38% [16/42]). No intervention reported refining theory, either by adding or removing theoretical constructs or by specifying that the interrelationships between theoretical constructs should be changed. The mean total theory use score was 4.4 (SD 5.43) out of a possible 22, which indicates that typically studies are not extensively reporting theory use in intervention development and evaluation. Multimedia Appendix 2 reports the 18 different theories or models mentioned and by which studies. The most frequently mentioned were motivational interviewing theory (38% [8/21]), transtheoretical model (29% [6/21]), and social norms theory (29% [6/21]).

### Association Between Reporting of Theory Use and Intervention Effectiveness

**Figure 2:**
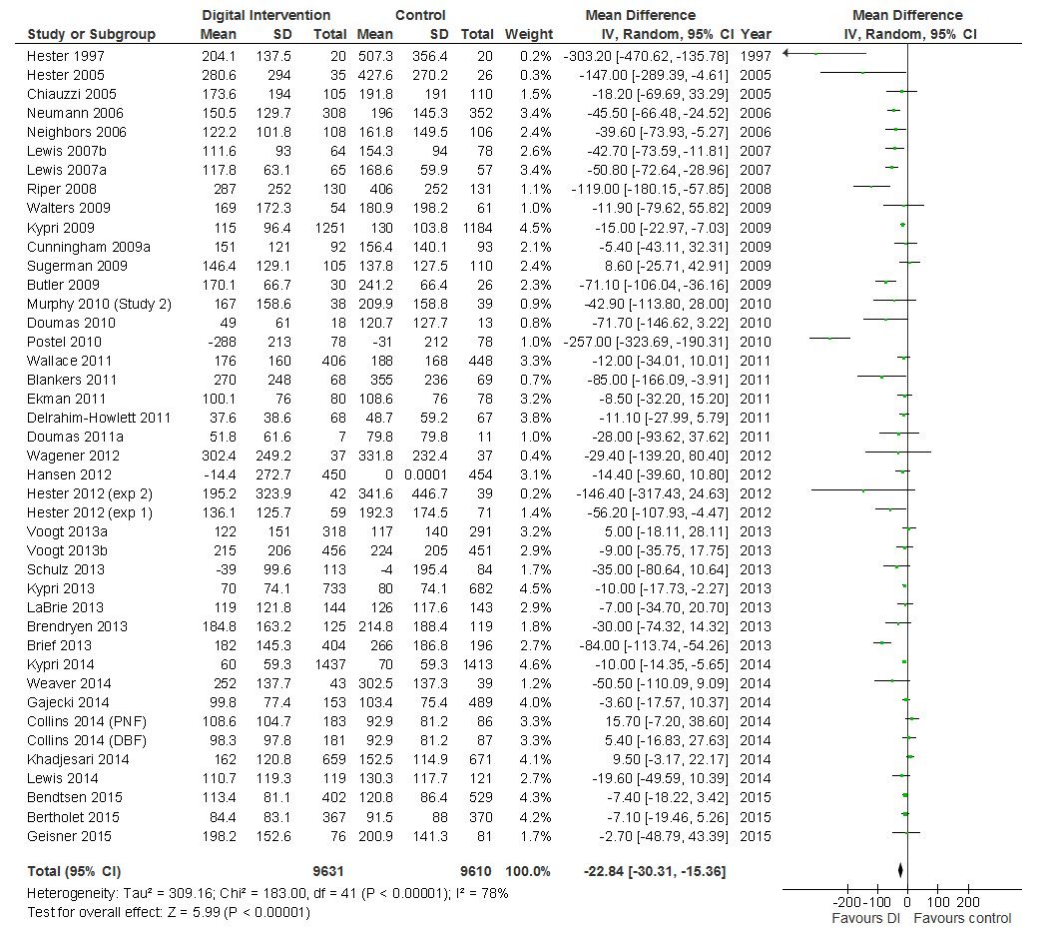
Digital intervention versus control-quantity of drinking (g/week), based on longest follow-up (reproduced from the main Cochrane review).

The primary meta-analysis in the Cochrane review found that participants randomized to a digital intervention drank 22.8 (95% CI 15.4-30.3) grams of alcohol per week less than controls [42], the equivalent of about 3 standard UK units of alcohol or 1.7 standard drinks in the United States. There was a significant proportion of the residual variation attributable to between study heterogeneity (I^2^=77.6%, *P*<.001; see Figure 2), which could potentially be explained by study-level covariates.

**Table 1:** Number of studies in which items on the theory coding scheme are present.

Theory coding scheme item (item number)		Item description [19]	Studies where item coded as present, n (%)
Theory or model of behavior mentioned (I1)		Models or theories that specify relations among variables to explain or predict behavior are mentioned even if the intervention is not based on this theory	21 (50)
Targeted construct mentioned as predictor of behavior (I2)		“Targeted” construct refers to a psychological construct that the study intervention is hypothesized to change	17 (40)
Intervention based on single theory (I3)		The intervention is based on a single theory (rather than a combination of theories or theory and predictors)	9 (21)
Theory or predictors used to select recipients for the intervention (I4)^a^		Participants were screened or selected based on achieving a particular score or level on a theory-relevant construct or predictor	0 (0)
Theory or predictors used to select or develop intervention techniques (I5)		The intervention is explicitly based on a theory or predictor or combination of theories and predictors	16 (38)
Theory or predictors used to tailor intervention techniques to recipients (I6)^a^		The intervention differs for different subgroups that vary on a psychological construct or predictor at baseline	3 (7)
All intervention techniques are explicitly linked to at least one theory-relevant construct or predictor (I7)		Each intervention technique is explicitly linked to at least one theory-relevant construct or predictor	6 (14)
At least one, but not all, of the intervention techniques are explicitly linked to at least one theory-relevant construct or predictor (I8)		At least one, but not all, of the intervention techniques are explicitly linked to at least one theory-relevant construct or predictor	11 (26)
Group of techniques are linked to a group of constructs or predictors (I9)^a^		A cluster of techniques is linked to a cluster of constructs or predictors	2 (5)
All theory-relevant constructs or predictors are explicitly linked to at least one intervention technique (I10)		Every theoretical construct within a state theory, or every stated predictor, is linked to at least one intervention technique	7 (17)
At least one, but not all, of the theory-relevant constructs or predictors are explicitly linked to at least one intervention technique (I11)		At least one, but not all, of the theoretical constructs within a stated theory or at least one, but not all, of the stated predictors (see I5) are linked to at least one intervention technique	10 (24)
Theory-relevant constructs are measured: post intervention (I12a)		At least one construct of theory (or predictor) mentioned in relation to the intervention is measured post intervention	12 (29)
Theory-relevant constructs are measured: post and pre intervention (I12b)		At least one construct of theory (or predictor) mentioned in relation to the intervention is measured pre and post intervention	10 (24)
Changes in measured theory-relevant constructs or predictors (I13)		The intervention leads to significant change in at least one theory-relevant construct or predictor (vs control group) in favor of the intervention	8 (19)
**Mediational analysis of constructs or predictors (I14):**			
	Mediator predicts the dependent variable (I14a)	Mediator predicts dependent variable, or change in mediator leads to change in dependent variable	6 (14)
	Mediator predicts dependent variable, controlling for the independent variable (I14b)^a^	Mediator predicts dependent variable when controlling for independent variable	3 (7)
	Intervention does not predict the dependent variable when controlling the independent variable (I14c)	Intervention does not predict dependent variable when controlling for mediator	4 (10)
	Mediated effect is statistically significant (I14d)	Mediated effect is statistically significant	6 (14)
	Results discussed in relation to theory (I15)	Results are discussed in terms of the theoretical basis of the intervention	12 (29)
	Appropriate support for theory (I16)	Support for the theory is based on appropriate mediation, or refutation of the theory is based on obtaining appropriate null effects (ie, changing behavior without changing the theory-relevant constructs)	7 (17)
	Results used to refine theory: adding or removing constructs to the theory (I17a)^a^	Authors attempt to refine the theory upon which the intervention was based by adding or removing constructs to the theory	0 (0)
	Results used to refine theory: specifying that the interrelationships between the theoretical constructs should be changed (I17b)^a^	Authors attempt to refine the theory upon which the intervention was based by specifying that the interrelationships between the theoretical constructs should be changed and spelling out which relationships should be changed	0 (0)

^a^Present in <10% of studies, so not included in the meta-regression analyses.

**Table 2:** Descriptive statistics for categories of theory use.

Theory coding scheme categories (category number)	Category description (what the items in each category assess) [19]	Items included	Maximum score	Mean (SD)	Studies scoring ≥1, N
Reference to underpinning theory (C1)	Stated or suggested, rather than demonstrated theoretical base	1, 2, 3	3	1.1 (1.23)	20
Targeting of relevant theoretical constructs (C2)	Whether evidence was provided that a targeted theoretical construct predicted behavior, whether theory or predictors were explicitly used for designing the intervention, and the extent to which the intervention targets particular theory-relevant constructs	2, 5, 6, 7, 8, 9, 10, 11	8	2.0 (2.43)	17
Using theory to select recipients or tailor interventions (C3)	Whether theory was used to select participants likely to benefit from the intervention, or to tailor the intervention to the needs of a particular individual	4, 6	2	0.1 (0.26)	2
Measurement of constructs (C4)	Whether the relevant theory-based constructs or predictors have been measured	12a, 12b	2	0.5 (0.86)	11
Testing of theory: mediation effects (C5)	Whether theoretical constructs are measured, whether the intervention changes the theoretical constructs, and whether these changes explain the effect	12a, 12b, 13, 14a, 14b, 14c, 14d, 15, 16	9	1.6 (2.83)	14
Refining theory (C6)^a^	Whether the results of evaluating theory-based interventions are used to refine theory	17a, 17b	2	—	—
Total use of theory	—	All items	22	4.4 (5.43)	20

^a^No score >0 for any studies, so not included in the meta-regression analyses.

#### Unadjusted Associations Between Reporting of Theory Use and Intervention Effectiveness

The unadjusted associations between reported theory use and intervention effectiveness are reported in Table 3. The TCS items, category scores, and total use of theory were entered as covariates in the analyses. Seven TCS covariates were not included in these analyses as they were either present in <10% of studies (items 4, 6, 9, 14b, 17a, and 17b) or had a composite score of 0 (category 6). The results indicated that the TCS covariates explained little of the heterogeneity, and no significant associations with intervention effectiveness were detected.

The Bayes factors derived from the reported theory use are reported in Table 3 and indicated that the majority of these data were insensitive to detect an effect. The TCS item of “Changes in measured theory-relevant constructs or predictor” had a Bayes factor greater than 3 (BF=3.50), which indicates moderate evidence for an association with intervention effectiveness. Seven TCS covariates had a Bayes factor of less than 0.33, indicating moderate evidence for no association between the item and intervention effectiveness (“targeted construct mentioned as predictor of behavior” BF=0.22; “theory or predictors used to select or develop intervention techniques” BF=0.27; “at least one, but not all, of the intervention techniques are explicitly linked to at least one theory-relevant construct or predictor” BF=0.23; “at least one, but not all, of the theory-relevant constructs or predictors are explicitly linked to at least one intervention technique” BF=0.30; “reference to underpinning theory” BF=0.12; “testing of theory: mediation effects” BF=0.24; and “total use of theory” BF=0.05). One TCS covariate had a Bayes factor of less than 0.10, indicating strong evidence for no association between the item and intervention effectiveness (“targeting of relevant theoretical constructs” BF=0.06).

**Table 3:** Unadjusted meta-regression analyses for the individual theory coding items, six categories of theory use, and use of theory scores.

Theory coding scheme covariates (item or category number)	B (SE)	*P* value	95% CI	Adjusted R^2^ (%)	I^2^ (%)	Bayes factor
Theory or model of behavior mentioned (I1)	9.73 (14.63)	.51	−19.84 to 39.31	−4.90	78.09	0.36
Targeted construct mentioned as predictor of behavior (I2)	24.17 (14.09)	.09	−4.30 to 52.64	2.27	78.13	0.22
Intervention based on single theory (I3)	12.92 (17.60)	.47	−22.64 to 48.49	−4.44	78.08	0.40
Theory or predictors used to select or develop intervention techniques (I5)	18.25 (14.57)	.22	−11.20 to 47.69	−3.43	78.15	0.27
All intervention techniques are explicitly linked to at least one theory-relevant construct or predictor (I7)	−3.73 (19.91)	.85	−43.98 to 36.51	−4.86	76.50	0.73
At least one, but not all, of the intervention techniques are explicitly linked to at least one theory-relevant construct or predictor (I8)	26.39 (15.34)	.09	−4.60 to 57.39	10.54	77.49	0.23
All theory-relevant constructs or predictors are explicitly linked to at least one intervention technique (I10)	8.53 (19.81)	.67	−31.60 to 48.46	−5.82	78.14	0.51
At least one, but not all, of the theory-relevant constructs or predictors are explicitly linked to at least one intervention technique (I11)	18.79 (15.99)	.25	−13.54 to 51.11	−3.45	78.15	0.30
Theory-relevant constructs are measured: post intervention (I12a)	−14.67 (15.81)	.36	−46.62 to 17.28	1.42	76.37	1.18
Theory-relevant constructs are measured: post and pre intervention (I12b)	−13.78 (16.88)	.42	−47.90 to 20.33	−1.67	76.94	1.09
Changes in measured theory-relevant constructs or predictor (I13)	−33.04 (17.48)	.07	−68.37 to 2.28	16.92	74.82	3.50
Mediational analysis of constructs or predictors: mediator predicts the dependent variable (I14a)	−7.77 (20.24)	.70	−48.68 to 33.15	−3.13	76.43	0.84
Mediational analysis of constructs or predictors: intervention does not predict the dependent variable when controlling the independent variable (I14c)	−21.88 (24.11)	.37	−70.61 to 26.86	4.48	75.41	1.29
Mediational analysis of constructs or predictors: mediated effect is statistically significant (I14d)	−7.77 (20.24)	.70	−48.68 to 33.14	−3.13	76.43	0.84
Results discussed in relation to theory (I15)	1.59 (16.08)	.92	−30.91 to 34.08	−6.81	77.35	0.54
Appropriate support for theory (I16)	−8.73 (19.43)	.66	−48.01 to 30.55	−2.11	76.33	0.87
Reference to underpinning theory (C1)	7.19 (5.89)	.23	−4.72 to 19.10	−1.55	78.08	0.12
Targeting of relevant theoretical constructs (C2)	3.94 (2.97)	.19	−2.06 to 9.93	−4.08	78.12	0.06
Using theory to select recipients or tailor interventions (C3)	13.30 (27.27)	.63	−41.81 to 68.42	−7.21	77.67	0.60
Measurement of constructs (C4)	−7.58 (8.41)	.37	−24.58 to 9.42	0.19	76.61	0.79
Testing of theory: mediation effects (C5)	−2.09 (2.53)	.41	−7.20 to 3.02	2.29	75.71	0.24
Total use of theory	0.39 (1.37)	.78	−2.38 to 3.15	−7.46	77.58	0.05

**Table 4:** Adjusted meta-regression analysis for the covariates with a meaningful association with effect size in unadjusted models.

Theory coding scheme covariates (item number)	B (SE)	*P* value	95% CI	Variance inflation factor	Bayes factor
Targeted construct mentioned as predictor of behavior (I2)	50.82 (21.00)	.02	8.31-93.34	2.98	0.24
At least one, but not all, of the intervention techniques are explicitly linked to at least one theory-relevant construct or predictor (I8)	−12.19 (20.71)	.56	−54.12 to 29.74	2.37	0.98
Changes in measured theory-relevant constructs or predictor (I13)	−61.41 (19.42)	.003	−100.71 to −22.10	1.45	23.71

#### Adjusted Associations Between Reporting of Theory Use and Intervention Effectiveness

An adjusted model was conducted entering the covariates that had a modest (albeit nonsignificant) association with effect size (B>23) in the unadjusted models (item 2, item 8, and item 13) and are reported in Table 4. The adjusted model had little effect on the degree of heterogeneity identified in the primary meta-analysis (I^2^=74.3% and adjusted R^2^=32.93%). The adjusted model produced two significant associations between TCS covariate and intervention effectiveness (item 2: “targeted construct mentioned as predictor of behavior” [B=50.82, 95% CI 8.31-93.34, *P*=.02] and item 13: “changes in measured theory-relevant constructs or predictor” [B=−61.41, 95% CI −100.71 to −22.10, *P*=.003]). However, these are difficult to interpret in the absence of any significant associations in the unadjusted models and that the pattern of results is not robust to standardized effect sizes or slight changes to the inclusion of studies.

## Discussion

### Principal Findings

There is limited reporting of theory use in the development or evaluation of digital interventions to reduce hazardous and harmful alcohol consumption. Half of the studies in this review did not make any reference to theory, and only a third of studies reported using theory to develop the intervention. No study reported using their results to refine the theory.

No significant associations were detected in the unadjusted models between the reporting of theory use and intervention effectiveness, though the meta-regression had limited power to detect modest associations [45], and any associations were likely to be small given the substantial heterogeneity in this literature. The data underlying the majority of null findings were found to be insensitive to distinguish an effect by calculating Bayes factors; however, there was moderate or strong evidence that eight TCS covariates are not associated with intervention effectiveness in this context. Insofar that a researcher believed smaller effect sizes were important, then it is likely these data would be judged as insensitive rather than supporting the null hypothesis. Despite failing to find evidence of a significant association, there was moderate evidence from the Bayes factor calculation that the item “changes in measured theory-relevant constructs or predictor” is associated with intervention effectiveness, which warrants further investigation. The adjusted model included three TCS covariates from the unadjusted models, and two of these had significant associations, though these results are difficult to interpret in the absence of significant results in the unadjusted models and that the pattern of results is not robust to standardized effect sizes.

### Comparison With Prior Work

The findings from this study differ from another recent review of studies assessing the association between theory use and effectiveness of computer-delivered alcohol interventions in the general population rather than our population of those with excessive or problematic drinking [14]. This difference is probably because of the different sample of studies included in each review: this review searched more databases though excluded a greater number of studies (eg, if follow-up was less than 1 month or participants were not screened and so not necessarily a hazardous or harmful drinker). This resulted in both reviews including unique studies as well as some common to both reviews. Another potential reason for the difference in findings is the way in which the TCS was used. In this review, all of the items relating to the reporting of theory use in intervention development and evaluation were used (excluding two relating to methodological issues), whereas in the Black et al review [14], only items relating to intervention development and participant selection (the first 11 items) were used.

### Limitations

Our results should be treated cautiously as the majority of null findings were insensitive to distinguish an effect, and the meta-regression was underpowered, which is a function of the available literature and methodology. However, it is important to have a starting point for collating evidence, and this meta-analysis can be updated as new literature emerges. It is estimated that more than 200 studies are required for 80% power to detect modest associations [45]. Once this level of power is reached, then additional analyses investigating associations between type of theory and intervention effectiveness may be insightful. A limitation of meta-regressions is that study characteristics can be highly correlated, which causes issues with multicollinearity [50]. However, in the adjusted model, the variance inflation factor statistics were less than three for each covariate, indicating that multicollinearity was not a major issue.

The composite scores calculated were crude measures that gave all items in the TCS equal weight, so were not necessarily the most accurate representation. However, the methodology used was the best tool currently available for assessing the reporting of theory use and quantifying its extent.

A limitation of the available literature was that only the *reporting* of theory use could be coded. This makes our findings difficult to interpret, as a lack of reporting of theory use in the published study does not necessarily equate to a lack of use of theory. Therefore, any inconsistent reporting of theory use between studies could have led to misclassification of studies, which cannot be accounted for. Future research could assess whether authors of the published studies code their studies using the TCS differently and whether this is associated with intervention effectiveness. This highlights the need for improvement in the way in which theory use is currently reported. The TCS can also be used as a checklist for researchers to use when reporting how theory was used, which would clarify whether theory had not been used or not been reported, and could be included as supplementary material alongside the trial evaluation paper. As the literature grows, a future meta-analysis including a larger number of studies should assess whether study characteristics moderate associations between theory use and intervention effectiveness.

### Future Research

A large number of behavior change theories exist [15], but only a relatively small number were used. The transtheoretical model was one of the most frequently used, despite lacking empirical support [51]. An absence of studies using their results to refine theories and, therefore, contribute to theory development was identified. Current behavior change theories are mainly based on limited static measures. Future research could study whether the development of digital interventions is better suited to dynamic, temporally sensitive theories [52]. The evaluation of digital interventions could help to develop this type of theory: the underpinning technology can often collect comprehensive data reflecting an individual’s behavior over time and in different settings and contexts [52-54].

### Conclusions

In sum, a lack of evidence was found that the reporting of theory use was associated with the substantial heterogeneity in effect between digital interventions for alcohol reduction. Limitations render the data and literature insensitive to answer the more general and important question of whether systematic use of a good and appropriate theory improves intervention effectiveness. Digital interventions provide an excellent opportunity to improve our understanding of behavior and, therefore, to develop dynamic, temporally sensitive behavior change theories [52-54]. However, no existing studies reported using their results to refine theory. This paper highlights the need for clearer selection, application, and reporting of theory use in the development and evaluation of digital behavior change interventions.

## Abbreviations

AUDIT: Alcohol Use Disorders Identification Test
BF: Bayes factor
IRR: interrater reliability
PABAK: prevalence-adjusted bias-adjusted kappa
TCS: theory coding scheme

## Appendix

Multimedia Appendix 1 References to studies included in meta-regression (reproduced from the main Cochrane review).

Multimedia Appendix 2 Matrix of which theories mentioned (item 1) for each study (n=21).
