# Supplementary material for: Reported Theory Use by Digital Interventions for Hazardous and Harmful Alcohol Consumption, and Association With Effectiveness: Meta-Regression
Source: J Med Internet Res. 2018 Feb 28;20(2):e69. doi: 10.2196/jmir.8807 (PMC5856921; doi:10.2196/jmir.8807)
Supplement: Multimedia Appendix 1 [file jmir_v20i2e69_app1.pdf]

References to studies included in this review (n=40, because 2 study reports contained 2 digital intervention arms each)

1. Bendtsen P, Bendtsen M, Karlsson N, et al. Online alcohol assessment and feedback for hazardous and harmful drinkers: Findings from the AMADEUS-2 randomized controlled trial of routine practice in Swedish universities. *J Med Internet Res* 2015;**17**:e170.

2. Bertholet N, Cunningham JA, Faouzi M, et al. Internet-based brief intervention for young men with unhealthy alcohol use: A randomized controlled trial in a general population sample. *Addiction* 2015;**110**(11):1735-1743.

3. Blankers M, Koeter MW, Schippers GM. Internet therapy versus internet self-help versus no treatment for problematic alcohol use: A randomized controlled trial. *J Consult Clin Psychol* 2011;**79**:330-41.

4. Brendryen H, Lund IO, Johansen AB, et al. Balance-a pragmatic randomized controlled trial of an online intensive self-help alcohol intervention. *Addiction* 2014;**109**:218-26.

5. Brief DJ, Rubin A, Keane TM, et al. Web intervention for OEF/OIF veterans with problem drinking and PTSD symptoms: A randomized clinical trial. *J Consult Clin Psychol* 2013;**81**:890-900.

6. Butler LH, Correia CJ. Brief alcohol intervention with college student drinkers: Face-to-face versus computerized feedback. *Psychology of Addictive Behaviors* 2009;**23**:163-7.

7. Chiauzzi E, Green TC, Lord S, et al. My student body: A high-risk drinking prevention web site for college students. *Journal of American College Health* 2005;**53**:263-74.

8. Collins SE, Kirouac M, Lewis MA, et al. Randomized controlled trial of web-based decisional balance feedback and personalized normative feedback for college drinkers. *J Stud Alcohol Drugs* 2014;**75**:982-92.

9. Cunningham JA, Wild TC, Cordingley J, et al. Twelve-month follow-up results from a randomized controlled trial of a brief personalized feedback intervention for problem drinkers. *Alcohol Alcohol* 2010;**45**:258-62.

10. Delrahim-Howlett K, Chambers CD, Clapp JD, et al. Web-based assessment and brief intervention for alcohol use in women of childbearing potential: A report of the primary findings. *Alcohol Clin Exp Res* 2011;**35**:1331-8.

11. Doumas DM, Haustveit T, Coll KM. Reducing heavy drinking among first year intercollegiate athletes: A randomized controlled trial of web-based normative feedback. *Journal of Applied Sport Psychology* 2010;**22**:247-61.

12. Doumas DM, Kane CM, Navarro B, et al. Decreasing heavy drinking in first-year students: Evaluation of a web-based personalized feedback program administered during orientation. *Journal of College Counseling* 2011;**14**:5-20.

13. Ekman DS, Andersson A, Nilsen P, et al. Electronic screening and brief intervention for risky drinking in Swedish university students - a randomized controlled trial. *Addict Behav* 2011;**36**:654-9.

14. Gajecki M, Berman AH, Sinadinovic K, et al. Mobile phone brief intervention applications for risky alcohol use among university students: A randomized controlled study. *Addict Sci Clin Pract* 2014;**9**:11.

15. Geisner IM, Varvil-Weld L, Mittmann AJ, et al. Brief web-based intervention for college students with comorbid risky alcohol use and depressed mood: Does it work and for whom? *Addict Behav* 2015;**42**:36-43.

16. Hansen AB, Becker U, Nielsen AS, et al. Internet-based brief personalized feedback intervention in a non-treatment-seeking population of adult heavy drinkers: A randomized controlled trial. *J Med Internet Res* 2012;**14**:e98.

17. Hester RK, Delaney HD. Behavioral self-control program for windows: Results of a controlled clinical trial. *J Consult Clin Psychol* 1997;**65**:686-93.

18. Hester RK, Delaney HD, Campbell W. The college drinker's check-up: Outcomes of two randomized clinical trials of a computer-delivered intervention. *Psychology of Addictive Behaviors* 2012;**26**:1-12.

19. Hester RK, Squires DD, Delaney HD. The drinker's check-up: 12-month outcomes of a controlled clinical trial of a stand-alone software program for problem drinkers. *Journal of Substance Abuse Treatment* 2005;**28**:159-69.

20. Khadjesari Z, Freemantle N, Linke S, et al. Health on the web: Randomised controlled trial of online screening and brief alcohol intervention delivered in a workplace setting. *PLoS One* 2014;**9**:e112553.

21. Kypri K, Hallett J, Howat P, et al. Randomized controlled trial of proactive web-based alcohol screening and brief intervention for university students. *Arch Intern Med* 2009;**169**:1508-14.

22. Kypri K, McCambridge J, Vater T, et al. Web-based alcohol intervention for Maori university students: Double-blind, multi-site randomized controlled trial. *Addiction* 2013;**108**:331-8.

23. Kypri K, Vater T, Bowe SJ, et al. Web-based alcohol screening and brief intervention for university students: A randomized trial. *Jama* 2014;**311**:1218-24.

24. Labrie JW, Lewis MA, Atkins DC, et al. RCT of web-based personalized normative feedback for college drinking prevention: Are typical student norms good enough? *J Consult Clin Psychol* 2013;**81**:1074-86.

25. Lewis MA, Neighbors C. Optimizing personalized normative feedback: The use of gender-specific referents. *J Stud Alcohol* 2007;**68**:228-37.

26. Lewis MA, Neighbors C, Oster-Aaland L, et al. Indicated prevention for incoming freshmen: Personalized normative feedback and high-risk drinking. *Addict Behav* 2007;**32**:2495-508.

27. Lewis MA, Patrick ME, Litt DM, et al. Randomized controlled trial of a web-delivered personalized normative feedback intervention to reduce alcohol-related risky sexual behavior among college students. *Journal of Consulting and Clinical Psychology* 2014;**82**:429–40.

28. Murphy JG, Dennhardt AA, Skidmore JR, et al. Computerized versus motivational interviewing alcohol interventions: Impact on discrepancy, motivation, and drinking. *Psychology of Addictive Behaviors* 2010;**24**:628-39.

29. Neighbors C, Lewis MA, Bergstrom RL, et al. Being controlled by normative influences: Self-determination as a moderator of a normative feedback alcohol intervention. *Health Psychol* 2006;**25**:571-9.

30. Neumann T, Neuner B, Weiss-Gerlach E, et al. The effect of computerized tailored brief advice on at-risk drinking in subcritically injured trauma patients. *J Trauma* 2006;**61**:805-14.

31. Postel MG, de Haan HA, ter Huurne ED, et al. Effectiveness of a web-based intervention for problem drinkers and reasons for dropout: Randomized controlled trial. *J Med Internet Res* 2010;**12**:e68.

32. Riper H, Kramer J, Smit F, et al. Web-based self-help for problem drinkers: A pragmatic randomized trial. *Addiction* 2008;**103**:218-27.

33. Schulz DN, Candel MJ, Kremers SP, et al. Effects of a web-based tailored intervention to reduce alcohol consumption in adults: Randomized controlled trial. *J Med Internet Res* 2013;**15**:e206.

34. Sugarman, DE. Web-based alcohol feedback intervention for heavy drinking college students: does drinking control strategy use mediate intervention effects?. PhD Thesis, Syracuse University 2009.

35. Voogt CV, Kleinjan M, Poelen EAP, et al. The effectiveness of a web-based brief alcohol intervention in reducing heavy drinking among adolescents aged 15-20 years with a low educational background: A two-arm parallel group cluster randomized controlled trial. *BMC Public Health* 2013;**13**:11.

36. Voogt CV, Poelen EA, Kleinjan M, et al. The effectiveness of the 'What Do You Drink' web-based brief alcohol intervention in reducing heavy drinking among students: A two-arm parallel group randomized controlled trial. *Alcohol Alcohol* 2013;**48**:312-21.

37. Wagener TL, Leffingwell TR, Mignogna J, et al. Randomized trial comparing computer-delivered and face-to-face personalized feedback interventions for high-risk drinking among college students. *Journal of Substance Abuse Treatment* 2012;**43**:260-7.

38. Wallace P, Murray E, McCambridge J, et al. On-line randomized controlled trial of an internet based psychologically enhanced intervention for people with hazardous alcohol consumption. *PLoS ONE* 2011;**6**:e14740.

39. Walters ST, Vader AM, Harris TR, et al. Dismantling motivational interviewing and feedback for college drinkers: A randomized clinical trial. *J Consult Clin Psychol* 2009;**77**:64-73.

40. Weaver CC, Leffingwell TR, Lombardi NJ, et al. A computer-based feedback only intervention with and without a moderation skills component. *Journal of Substance Abuse Treatment* 2014;**46**:22-8.
