# Supplementary material for: Reported Theory Use by Digital Interventions for Hazardous and Harmful Alcohol Consumption, and Association With Effectiveness: Meta-Regression
Source: J Med Internet Res. 2018 Feb 28;20(2):e69. doi: 10.2196/jmir.8807 (PMC5856921; doi:10.2196/jmir.8807)
Supplement: Multimedia Appendix 2 [file jmir_v20i2e69_app2.pdf]

Multimedia Appendix 1. Matrix of which theories mentioned (item 1) for each study (n=21).

First author & year of publication	No. of theories	Total theory use score	Motivational Interviewing Theory	Social Norms Theory	Transtheoretical Model	I-change Model	Social Comparison Theory	Social Impact Theory	Theory of Planned Behaviour	Self-Regulation Theory	Social Cognitive Theory	Social Identity Theory	Social Learning Theory	Theory of Social Influence	Cognitive-Behavioural Theory	Decision-Making Theory	Expectancy Theory	Health Belief Model	Problem Behaviour Theory	Social Determination Theory
Brendryen 2013	3	6			X					X	X									
Collins 2014 (DBF)	3	6	X		X											X				
Collins 2014 (PNF)	1	6											X							
Doumas 2010	1	15		X																
Gajecki 2014	1	7							X											
Geisner 2015	1	8		X																
Hansen 2012	1	8								X										
Kypri 2014	1	1										X								
LaBrie 2013	3	12		X			X	X												
Lewis 2007a	4	16		X			X	X				X								
Lewis 2007b	3	14		X			X	X												
Lewis 2014	2	15		X									X							
Murphy 2010 (study 2)	1	12	X																	
Neighbors 2006	1	16																		X
Postel 2010	2	4	X		X															
Schulz 2013	5	9			X	X			X		X							X		
Sugarman 2009	3	6	X												X				X	
Voogt 2013a	3	5	X			X								X						
Voogt 2013b	5	8	X		X	X			X					X						
Wallace 2011	2	7	X		X															
Weaver 2014	2	2	X														X			
Number of studies:			8	6	6	3	3	3	3	2	2	2	2	2	1	1	1	1	1	1
